# Increased Phenolic Content and Enhanced Antioxidant Activity in Fermented Glutinous Rice Supplemented with Fu Brick Tea

**DOI:** 10.3390/molecules24040671

**Published:** 2019-02-14

**Authors:** Xiao Xu, Wenxiu Hu, Siduo Zhou, Chuanhai Tu, Xiudong Xia, Juanmei Zhang, Mingsheng Dong

**Affiliations:** 1 College of Food Science and Technology, Nanjing Agricultural University, Nanjing, Jiangsu 210095, China; xiaolexu@126.com (X.X.); wenxiu.hu94@outlook.com (W.H.); zhousiduo@126.com (S.Z.); 2017208026@njau.edu.cn (C.T.); 2 Institute of Agro-Product Processing, Jiangsu Academy of Agricultural Sciences, Nanjing, Jiangsu 210014, China; 86084056@163.com; 3 College of Pharmaceutical, Henan University, Kaifeng, Henan 475004, China

**Keywords:** fermented glutinous rice, Fu brick tea, antioxidant activities, DNA damage protection, phenolic compounds, sensory evaluation

## Abstract

Glutinous rice-based foods have a long history are consumed worldwide. They are also in great demand for the pursuit of novel sensory and natural health benefits. In this study, we developed a novel fermented glutinous rice product with the supplementation of Fu brick tea. Using in vitro antioxidant evaluation and phenolic compounds analysis, fermentation with Fu brick tea increased the total phenolic content and enhanced the antioxidant activity of glutinous rice, including scavenging of 1,1-Diphenyl-2-picryl-hydrazyl (DPPH) radical, 2,2′-azino-bis-3-ethylbenzthiazoline-6-sulphonic acid (ABTS) radical, and hydroxyl radical, ferric-reducing antioxidant power, and ferric ion reducing power and iron chelating capability. Besides, compared with traditional fermented glutinous rice, this novel functional food exhibited a stronger activity for protecting DNA against hydroxyl radical-induced oxidation damage. Quantitative analysis by HPLC identified 14 compounds covering catechins and phenolic acids, which were considered to be positively related to the enhanced antioxidant capability. Furthermore, we found that 80% ethanol was a suitable extract solvent compared with water, because of its higher extraction efficiency and stronger functional activities. Our results suggested that this novel fermented glutinous rice could serve as a nutraceutical food/ingredient with special sensory and functional activities.

## 1. Introduction

Fu brick tea (FBT), a popular beverage processed through the post-fermentation of *Camellia sinensis* L. (family Theaceae) leaves, is rich in antioxidant polyphenols like catechins, theaflavins, and other bioactive compounds [1]. Theaflavins, thearubigins, and other conjugated phenolics of FBTs are considered as one of the most important factors that are associated with color characteristics, astringency flavor, and antioxidant activity [2]. The health benefits of FBT have been found in various fields such as protection against cancer, pathogenic bacteria, diabetes, and cardiovascular and neurological disorders [1,2,3]. FBT is welcomed in many countries, especially in west Asia, in which people intake high-calorie and high-carbohydrate diets. This is because FBT has been found to participate in controlling carbohydrate metabolism and the absorption balance of cholesterol and fat [4]. Besides, a higher dietary intake of phenolic compounds has been certified to have positive effects on lowering the risk of oxidation-induced chronic diseases [5]. Therefore, it is rational to incorporate FBT into starch-rich foods to produce a novel functional food. The supplementation of FBT could increase its phenolic content and enhance its health benefits, along with introducing novel sensory properties.

Rice-based fermented foods are indispensable and are consumed widely in Asia as traditional staple foods and drinks, such as Korean Koji, Indian Dhokla, and Chinese rice wine [6,7]. Among them, fermented glutinous rice (FGR) is in great demand, and its sales went up from 8.96 billion to 19.83 billion yuan in China in over years (from 2010 to 2016) [8]. Over the past few decades, fermented glutinous rice has risen popularity outside Asia, owing to its unique flavor and its nutritional value. As an excellent source of natural antioxidants, abundant phenolic acids in fermented glutinous rice are liberated from conjugated phenolic compounds in the raw material by fermentation, but the content of phenolic compounds is still lower than juice and tea beverages [9]. Therefore, in this study, FBT was supplemented with traditional fermented glutinous rice for elevating the content of natural phenolic compounds.

Tea phenolic compounds have a diverse range of solubilities in various extraction solvents (e.g., water, ethanol, and other organic solvents) [10]. The fermentation progress is equivalent to an extraction progress for tea phenolics, in which the juice of fermented glutinous rice, which is a mixed solution of water and organic constituents, is produced from raw materials. Moreover, tea catechins are stable in an acidic system (pH < 4) [11]. Previous studies have found that many acids are produced during the fermentation process of fermented glutinous rice, such as acetic acid, lactic acid, and other organic acids [12]. Hence, in the system of fermented glutinous rice, an acidic system was provided for benefit of the stability of phenolic compounds in FBT.

Polyphenols and other macromolecular chemicals bound with polysaccharide and cellulose can be biotransformed by microorganisms during fermentation [6,7]. Also, the biotransformation effect is adequate for the hydrolyzation of polyphenols in FBT. The release of polyphenols has a positive effect on their solubilities and bioavailabilities and contributes to the enhancement of functional activities. Xu et al. stated that fermentation changed the proportion of phenolic compounds, and it had a positive effect on the antioxidant activities of rice [9]. Huang et al. reported that a new functional food combining red yeast rice with *Puerariae Radix* had an increased total phenolic content and enhanced antioxidant activity after mixed fermentation [13]. Wang et al. produced a novel bio-tofu by adding Fu brick tea extract, and its antioxidant activity was improved [14]. In addition, the supplementation of natural phenolic compounds could promote the sensory and textural properties of food [15].

In this study, a novel fermented glutinous rice product was developed by mingling with Fu brick tea (FGR-FBT). The impacts of FBT addition on the total phenolic content, antioxidant activities, and the protection against DNA oxidation damage by hydrogen peroxide were evaluated in vitro. Besides, the major functional compounds in FGR-FBT were analyzed by high performance liquid chromatography (HPLC). Apart from the phenolic compounds and its functional benefits, influences on the color and eating quality of FGR were also evaluated. This study developed a new nutrition-rich glutinous rice-based food, which has potential for protecting living bodies against oxidative damage, as well as a range of oxidative damage-induced diseases. Furthermore, this study may stimulate further interest in using Fu brick tea as a supplement for the development of novel products with natural antioxidants.

## 2. Results and Discussion

### 2.1. Optimization of the Supplementation of FBT to produce FGR-FBT

**Figure 1:**
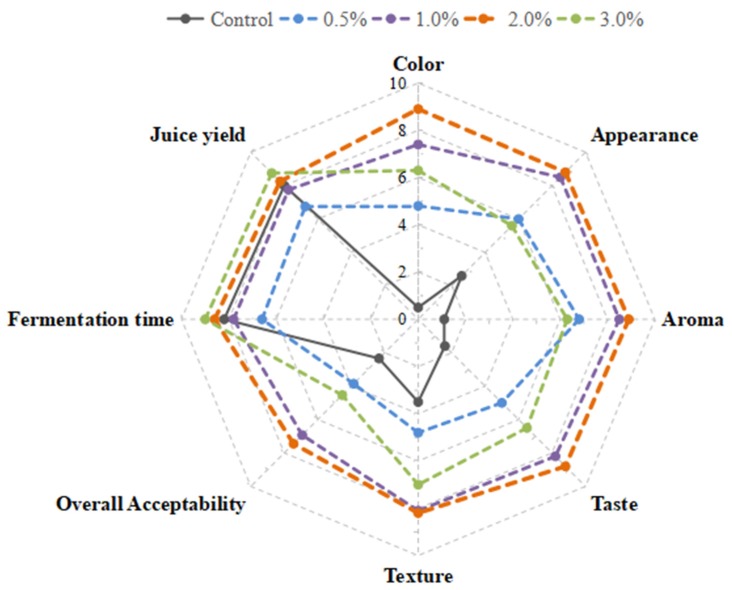
Proportion optimization of the supplementation of Fu brick tea in the fermentation of glutinous rice, analyzed by juice yield, fermentation time, sensory scores of color, appearance, aroma, taste, texture, and overall acceptability.

To optimize and finalize the additive content of FBT, various ratios of FBT powder supplementation (0.5%, 1%, 2%, 3%) were added to FGR and FGR-FBT for evaluation based on juice yield, fermentation time, and sensory properties. The juice yield of each sample was measured over the time period of fermentation, and the fermentation time was determined according to the terminal properties. As shown in Figure 1, FGR-2%FBT and FGR-3%FBT obtained higher scores on juice yield and fermentation time, compared with those of the control. The results suggested that FGR with 2% and 3% supplementation of FBT improved fermentation efficiency, shortened fermentation time, and increased the juice yield. Besides, the scores of colors, appearance, aroma/flavor, taste/mouth-feel and basic texture obtained by FGR-2%FBT were higher than FGR-3%FBT and others, and this indicated that a 2% supplementation of FBT had a positive effect from sensory evaluation, and it possessed the best sensory properties. Additionally, the higher sensory scores of FGR-1%FBT indicated its better sensory acceptability compared with the excessive tea-like color, heavy bitter and astringent taste of FGR-3%FBT. Nevertheless, FGR-1%FBT provided a lower juice yield and needed a longer fermentation time. Therefore, through a comprehensive analysis, 2% FBT supplementation was used in the formulation of the novel glutinous rice product for further study.

### 2.2. Sensory Evaluation

Sensory analysis was conducted for analyzing fermented glutinous rice products between control and phenolic-enriched formulas in the present study. FGR-FBT and FGR were evaluated on their appearance, aroma/flavor, taste/mouth-feel and textural properties by panelists, and the results are shown in Table 1. There were insignificant differences among samples in turbidity and irritant taste, and few differences between samples with adjacent FBT addictive proportions with the aspects of alcohol and acid aroma, sour taste, and full body, suggesting that these parameters were most dependent on fermentation and glutinous rice quality. On the contrary, other 10 sensory aspects displayed significant differences among different samples (*p* < 0.05) including lightness, color, tea and cereal aroma, bitterness, sweetness, astringency and aftertaste, granular feel, and continuation. Except for lightness, sweet taste and cereal aroma, the scores of the parameters for FGR-FBT dropped markedly when the FBT concentration was decreased. The supplementation of phenolic compounds, flavones, and alkaloids that was brought about by FBT resulted in an increased degree of bitterness, astringency, aftertaste, and granular sense. Moreover, the elevated tea aroma resulting from these compounds was accompanied by a reduction in cereal aroma, because the tea aroma diluted and dissipated the fragrance of the fermented glutinous rice. As a result, FGR-FBT was endowed with integrated herbal, fruity, and flower flavors, indicating the great potential of adding FBT at suitable concentrations to produce novel sensory properties in the glutinous rice product. Furthermore, the decreased lightness and increased tea-color were attributed to theaflavins, thearubigins, and other anthocyanin pigments in FBT. The altered tea-like color of fermented glutinous rice products may be an attractive property for consumers.

### 2.3. Total Phenolic Content 

**Figure 2:**
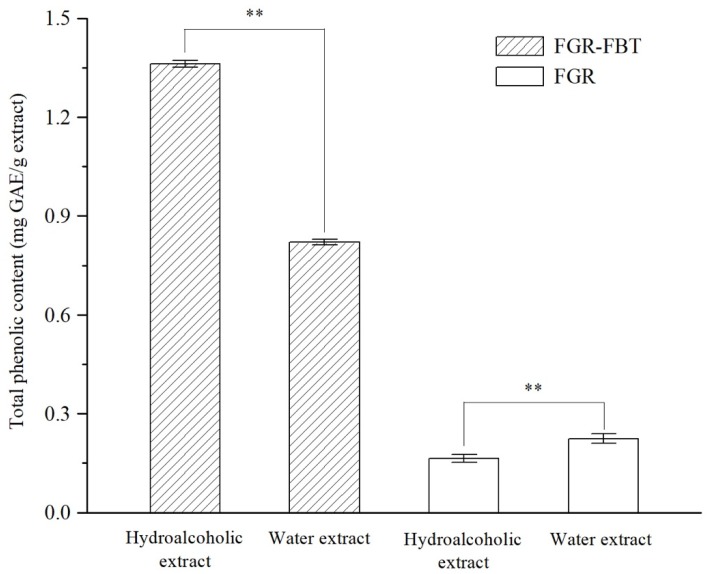
The total phenolic contents of hydroalcoholic extracts (80% ethanol extracts) and water extracts of fermented glutinous rice with Fu brick tea (FGR-FBT) and traditional fermented glutinous rice (FGR). ** shows significant differences between samples (*P* < 0.01).

Phenolic compounds in natural plants have attracted considerable attention with regard to their physiological functions in protecting human bodies against oxidation, ageing, cancer, and diabetes [3,9]. In most studies, different compositions of phenolic compounds extracted by different solvents differed in their bioactive properties [1]. In this study, FGR-FBT and FGR were extracted by water and 80% ethanol. The total phenolic contents were measured and compared between two samples using extractions by various solvent systems. As shown in Figure 2, the total phenolic content of FGR-FBT extracted by 80% ethanol was 1.362 ± 0.010 mg GAE/g extract, which was the highest, followed by a water extract of FGR-FBT (0.821 ± 0.009 mg GAE/g extract), FGR water extract (0.225 ± 0.015 mg GAE/g extract) and FGR hydroalcoholic extract (0.165 ± 0.012 mg GAE/g extract). The total phenolic content of FGR-FBT was significantly higher than FGR (*p* < 0.05), suggesting that the supplementation of FBT increased the total phenolic contents of the traditional fermented glutinous rice products. Furthermore, the results clearly demonstrated that 80% ethanol solvent was more efficient for extracting phenolic compounds from FGR-FBT, while the water system was more suitable for FGR. Although previous studies have reported that fermentation increased the phenolics of cereals [16,17], the supplementation of FBT affected the total phenolic content more directly and deeply. Thereby, developing glutinous rice products by adding FBT has great potential as phenolics-rich formulations.

### 2.4. HPLC Analysis of Phenolic Compounds

Analyses of individual phenolic compounds in FGR-FBT and FGR were performed by HPLC. According to previous studies, syringic acid, gallic acid, catechin, and some phenolics were found in fermented glutinous rice [18,19,20], but in this study, they were not detected in FGR extracts, possibly because their contents were lower than the detection limit. Due to the great disparity between FGR-FBT and FGR extracts, the phenolic content and several functional components from Fu brick tea became our focus in the HPLC analysis of FGR-FBT. Fourteen chemicals were analyzed, and the results were shown in Table 2. Among them, gallic acid was considered to be the principal phenolic acid, as it was present at concentrations of 81.30 ± 0.31 and 77.47 ± 0.33 μg/g respectively, in hydroalcoholic extract and water extract. As for the catechins, (−)-gallocatechin (GC) and (−)-epicatechin (EC) were dominant, at concentrations of 269.81 ± 2.77 and 152.98 ± 1.41 μg/g respectively in hydroalcoholic extract, and 255.97 ± 1.71 and 119.71 ± 1.72 μg/g respectively in water extract; this was followed by (−)-epigallocatechin gallate (EGCG), (−)-gallocatechin gallate (GCG), (−)-epigallocatechin (EGC), catechin (C), and (−)-epicatechin gallate (ECG). Zhang et al. reviewed the contents of catechins and gallic acid in different Chinese dark teas, where differences in phenolic constituents were exhibited [1]. In another study, ECG, EGCG and EC of aged fermented tea were found as dominant constituents, followed by EGC, GC, C, and GCG [21]. It was also regarded that the chemical constituents of tea were qualitatively and quantitatively changed, which resulted from the production of metabolites of catechins and phenolic acids by fermentation. Nevertheless, whatever the biotransformation carried out during the fermentation process, the large amount of catechins and phenolic acids would have a positive effect on physical health.

Other than phenolics, caffeine is thought to be one of the major functional compounds in teas that confers a protective activity against some diseases, including Parkinson’s disease [22]. There was a high concentration of caffeine, at 267.06 ± 0.54 μg/g from the hydroalcoholic extract and 272.46 ± 0.13 μg/g from the water extract, detected in FGR-FBT. As for another characteristic alkaloid, theobromine and theophylline were also quantified at 4.64 ± 0.17/1.43 ± 0.03 μg/g in the hydroalcoholic extract, and 9.80 ± 0.07/1.42 ± 0.02 μg/g in the water extract, respectively. As we all know, theobromine is the precursor to caffeine, which in turn is the precursor to theophylline. It has been certificated a moderate intake of these is associated with the stimulation of the nervous system, and an increase in blood pressure and heart rate [23]. Furthermore, 13.25 ± 0.10/13.18 ± 0.04 μg/g hydroalcoholic/water extracts of 3,4-dihydroxybenzaldehyde was detected in FGR-FBT, which was found to have antioxidant, anti-inflammatory and antitumor activities [24]. Also, 3,4-dihydroxybenzoic acid was detected in FGR-FBT, with similar beneficial effects [25].

The constituents of individual compounds displayed variations when extracted by various solvents with polarity differences. As shown in Table 2, theobromine and caffeine had significantly higher solubilities in the water extract than in the hydroalcoholic extract (*p* < 0.05), indicating that a water system is a suitable extraction method for alkaloids. Except for these, most phenolic compounds were obtained in greater amounts by extraction with 80% ethanol. Therefore, similar to the total phenolic content results, the content sum of catechins and phenolic acids in the hydroalcoholic extract was significantly larger than the water extract, suggesting that the production of ethanol during fermentation helped with the dissolution of phenolics. Also, 80% ethanol has the potential to extract functional chemicals in FGR-FBT for the development of nutraceuticals.

Additionally, the content sum of the phenolics as quantified by HPLC was much lower than the results by total phenolic content (TPC) analysis. Similar results have been reported by previous studies [26,27]. One of major reasons for a higher TPC than the actual value is that the Folin–Ciocalteu reagent for determining TPC could react with other non-phenolics by its lack of specificity, such as reducing sugar, amino acids, and peptides [13]. On the other hand, some phenolic compounds in trace amount and conjugated compounds were not detected by HPLC analysis, but were present according to TPC analysis.

### 2.5. Antioxidant Activities

**Figure 3:**
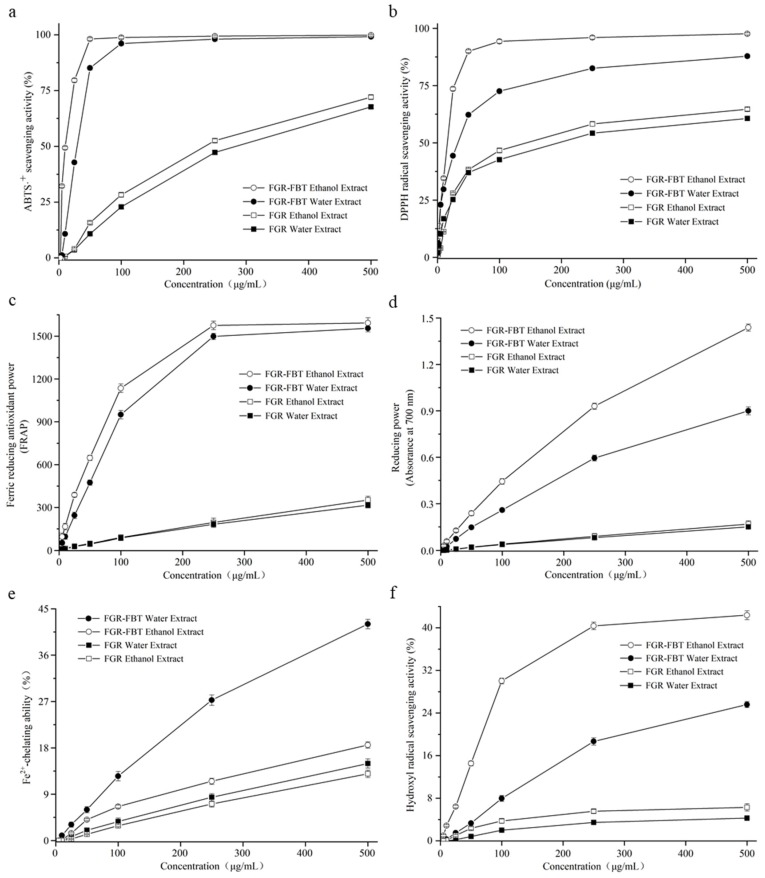
Antioxidant properties of hydroalcoholic extracts (80% ethanol extracts) and water extracts of fermented glutinous rice with Fu brick tea (FGR-FBT) and traditional fermented glutinous rice (FGR) evaluated by different methods: ABTS·^+^ (2,2′-azino-bis-3-ethylbenzthiazoline-6-sulphonic acid) radical-scavenging activity (**a**), DPPH (1,1-Diphenyl-2-picryl-hydrazyl) radical-scavenging activity (**b**), ferric-reducing antioxidant power (FRAP) (**c**), reducing power (**d**), Fe^2+^-chelating ability (**e**) and hydroxyl radical-scavenging activity (**f**).

Antioxidant activity refers to many aspects, and it can be influenced by many factors [13]. To make a comprehensive evaluation on the antioxidant effect of samples, it is necessary to carry out multiple methods based on their individual reaction principles. In this study, six measurement systems were utilized for complementary analysis, including the scavenging activities of ABTS·^+^ (2,2′-azino-bis-3-ethylbenzthiazoline-6-sulphonic acid), DPPH (1,1-Diphenyl-2-picryl-hydrazyl) radical and hydroxyl radical, ferric reducing antioxidant power, reducing power and chelating ability of ferrous ions. The antioxidant activities of the hydroalcoholic and water extracts of FGR and FGR-FBT are shown in Figure 3a–f and IC_50_ values are shown in Table 3.

#### 2.5.1. ABTS·^+^ and DPPH Radical-Scavenging Activities

ABTS·^+^ and DPPH radical-scavenging activities were utilized for evaluating the capability of samples to scavenge free radicals. As shown in Figure 3a,b, all the samples exhibited in a dose dependence manner whose enhanced activities were correlated with increased concentrations of samples. For instance, FGR-FBT extracted by water had 10.76 ± 0.49%, 85.15 ± 0.86%, and 98.09 ± 0.75% ABTS·^+^-scavenging activities at concentrations of 10 μg/mL, 50 μg/mL and 250 μg/mL, respectively. However, at the same concentration, the FGR-FBT hydroalcoholic extract displayed 49.30 ± 0.59%, 98.11 ± 0.55% and 99.44 ± 0.42% of scavenging ratios, suggesting stronger antioxidant activity from the hydroalcoholic extract compared with the water extract. It was also noted that FGR-FBT had free radical-scavenging activity that was comparable to vitamin C, where 50 μg/mL of hydroalcoholic extract and 100 μg/mL of water extract scavenged over 90% of ABTS·^+^ radical. As for FGR, only 0.63 ± 0.07%/0.61 ± 0.06%, 15.75 ± 0.55%/10.75 ± 0.59% and 52.58 ± 0.77%/47.28 ± 0.95% of ABTS·^+^ were scavenged by hydroalcoholic/water extracts at the same concentration. The results indicated that the ABTS·^+^ scavenging capability of FGR-FBT was much higher than that of FGR. It could also be confirmed by the lower IC_50_ values of 10.75 ± 0.24/30.67 ± 0.29 μg/mL exhibited by FGR-FBT hydroalcoholic/water extract, compared with 224.55 ± 3.63/257.71 ± 4.82 μg/mL of FGR.

The DPPH radical-scavenging activities of samples were similar to the ABTS·^+^ results. As shown in Figure 3b, the FGR-FBT hydroalcoholic/water extracts scavenged 93.14 ± 0.78%/72.56 ± 0.81% of DPPH radical at 500 μg/mL, which were 2.00- and 1.70-fold scavenging ratios than FGR respective extracts. At a low concentration, the difference of the antioxidant activity was broader between FGR and FGR-FBT. For example, the scavenging ratio of the FGR-FBT hydroalcoholic extract was 72.65 ± 0.81%, which was 1.64-, 2.59-, and 2.88-fold higher than that of the FGR-FBT water extract, and the FGR hydroalcoholic and water extracts, respectively.

#### 2.5.2. Ferric-Reducing Antioxidant Power and Reducing Power

The reducibility of the sample is related to its potential for reducing oxides, and thereby its exhibited antioxidant activities. This study accomplished ferric-reducing antioxidant power (FRAP) and reducing power assays to evaluate the reducibility of FGR-FBT and FGR. Similar to the results of the free radical-scavenging activities, the reduction potential was elevated with increased dosage, regardless whether 80% ethanol or water extracts were used. As shown in Figure 3c, FRAP was expressed as ferrous iron equivalent. A higher Fe(II) equivalent mirrored a stronger antioxidant activity. In the study, higher FRAP values of 1592.36 ± 37.11/1551.82 ± 25.98 μM Fe(II) were obtained respectively with FGR-FBT extracted using 80% ethanol/water and diluted into 500 μg/mL, while at the same concentration, 351.82 ± 25.98/315.45 ± 18.56 μM Fe(II), respectively, were detected in FGR hydroalcoholic and water extracts.

Similarly, Figure 3d shows the changed reducing power of samples with various concentrations. The IC_50_ values of FGR-FBT and FGR were 126.42 ± 3.83 and 1648.89 ± 8.19 μg/mL, respectively, in hydroalcoholic extract, while they were 265.61 ± 10.69 and 1654.89 ± 24.26 μg/mL, respectively, in water extract. The FRAP and reducing power results indicated that the reducibility of traditional fermented glutinous rice was significantly improved by the supplementation of Fu brick tea.

#### 2.5.3. Chelating Capability of Fe^2+^ and Hydroxyl Radical-Scavenging Activity

Another antioxidant system was performed by determining the capability of chelating Fe^2+^ and the hydroxyl radical-scavenging activity. It has been confirmed that ferrous iron (Fe^2+^) can react with hydrogen peroxide (H_2_O_2_) in the body to generate hydroxyl radicals that cause damage to proteins, nucleic acids, and phospholipids, thereby leading to cellular degeneration [28]. Iron chelation is an effective method of relieving exacerbated oxidative stress from the presence of excess iron [29]. The chelating capability of FGR was weaker than that of FGR-FBT (Figure 3e). Notably, water extracts maintained a stronger activity for chelating ferrous iron, compared with hydroalcoholic extracts. For example, the IC_50_ values of FGR-FBT were 498.43 ± 13.75 μg/mL (water extract) and 705.47 ± 55.92 μg/mL (hydroalcoholic extract). Similar results were presented for the values of FGR, which were respectively, 3.37- and 2.68-fold higher than that of FGR-FBT. This demonstrates that other chemicals besides phenolics that are soluble in water play crucial roles, such as functional oligosaccharides, peptides, and polysaccharides. Chen et al. reported that heteropolysaccharides in FBT had a protective effect on H_2_O_2_-induced oxidative injury [30].

In the evaluation on hydroxyl radical-scavenging activity (Figure 3f), with the effects of FGR-FBT and FGR, oxidative radicals were scavenged at a lower proportion compared with other free radicals. At 500 μg/mL, FGR-FBT hydroalcoholic/water extracts respectively displayed 43.34 ± 0.85%/25.59 ± 0.56% of hydroxyl radical-scavenging activities; while FGR showed 24.15 ± 0.56%/22.04 ± 0.66%, respectively. Even though the IC_50_ values of FGR-FBT were calculated by extrapolation from linear regression analysis, from a raised tendency in the dose-dependent system, it was possible that FGR-FBT scavenged over half of the hydroxyl radicals at a higher concentration.

All of the above results demonstrate that the antioxidant activities of fermented glutinous rice could be increased by the supplementation of Fu brick tea, which increases total phenolic content and the level of free soluble functional chemicals. Zhu et al. increased the antioxidant properties of Chinese steam bread by incorporating black tea [31]. Similarly, the addition of green tea powder into whole-wheat flour increased the antioxidant activity effectively [32]. Recently, the supplementation of functional foods or ingredients has become a hot topic. For instance, linseed addition enhanced the antioxidant activities of fresh noodles; purple grape skin flour and juice were used to enhance the nutritional quality of yogurts [33]; *Inula britannica* (family Daisy) flower extract fortified the antioxidant functions of Cheddar-type cheese [34]. In practice, the development of ‘natural’ foods has been paid more and more attention, that is, with minimal or no use of synthetic chemical compounds, but with natural and healthy ingredients. This study provided the basis for the better utilization of Fu brick tea as a functional addition, and promoted the development of novel types of fermented glutinous rice.

### 2.6. Inhibition of Hydroxyl Radical-Induced Supercoiled Plasmid DNA Strand Breakage

**Figure 4:**
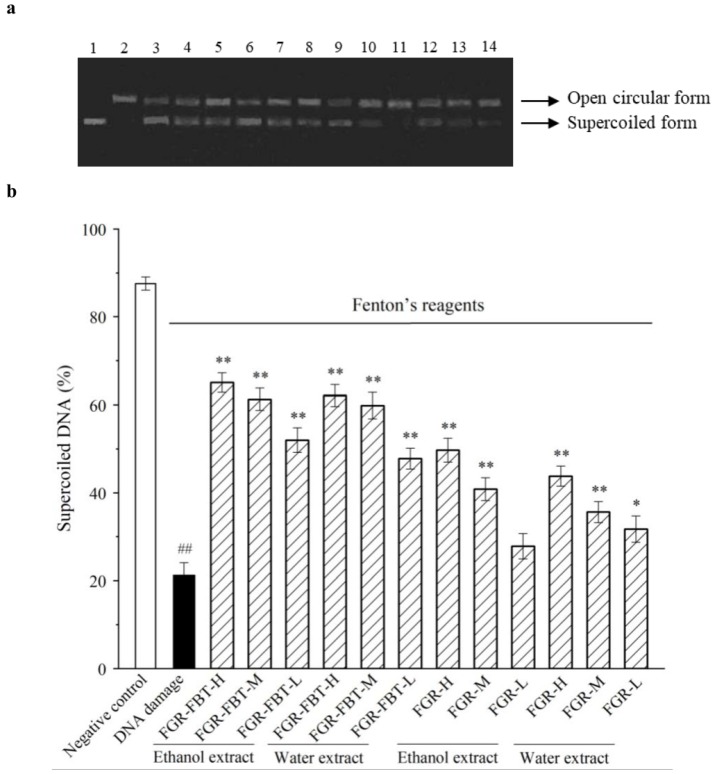
(**a**). DNA oxidative damage-protecting effect of hydroalcoholic extracts (80% ethanol extracts) and water extracts of fermented glutinous rice with Fu brick tea (FGR-FBT) and traditional fermented glutinous rice (FGR) against hydroxyl radical-induced DNA damage of pUC18; (**b**). Protective activities of samples against DNA damage induced by Fenton’s reagent, expressed by the percentage presence of the supercoiled DNA form. Lane 1: native pUC18 plasmid DNA (negative control); lane 2: DNA + Fenton’s reagent (DNA damage control); lane 3-5: DNA + Fenton’s reagent + FGR-FBR hydroalcoholic extract at high, middle, and low concentrations; lane 6-8: DNA + Fenton’s reagent + FGR-FBR water extract at high (H), middle (M), and low (L) concentrations; lane 9-11: DNA + Fenton’s reagent + FGR hydroalcoholic extract at H, M, and L concentrations; lane 12-14: DNA + Fenton’s reagent + FGR water extract at H, M, and L concentrations. The gels were visualized under a UV-transilluminator. H, M, and L concentrations are 400 μg/mL, 200 μg/mL, and 100 μg/mL of samples, respectively. ## indicates significant differences between the negative control and the DNA damage control (*P* < 0.01). * indicates significant differences between the samples and the DNA damage control (*P* < 0.05). ** indicates significant differences between the samples and the DNA damage control (*P* < 0.01).

DNA damage could be caused by oxygen species, including free radicals, hydroxyl radicals, and hydrogen peroxide [35]. In this study, hydroxyl radicals generated by the Fenton reaction were used for the oxidative damage of DNA, in which H_2_O_2_ was cleaved by electron transfer from ferrous ion according to the method of Xiao et al. [26]. The pUC18 plasmid DNA in supercoiled form (native form) was damaged by Fenton reagents and cleaved into open circular forms as the control, as shown in Figure 4a (lane 2). The DNA in native form without Fenton’s reagent and antioxidant additives is shown in Lane 1 as the negative control. As for hydroalcoholic and water extracts of FGR-FBT and FGR, a concentration range of 100–500 μg/mL was considered for the exhibition of good antioxidant activity. Thus, the extracts were evaluated for their protective activities of inhibiting supercoiled DNA strand scission at low, middle, and high concentrations of 100 μg/mL, 200 μg/mL, and 400 μg/mL respectively.

The percentages of the supercoiled DNA form in each reaction are shown in Figure 4b, and these were calculated based on the optical intensity of the gel band. A higher supercoiled DNA percentage indicated a lower intensity of oxidative forms (open circular forms), which also suggested a stronger activity of inhibiting DNA damage. Compared with 21% of supercoiled DNA in the control group, the inhibitive effect of DNA scission was strong, ranging from 49% to 67% supercoiled DNA displayed under the protection of 100–400 μg/mL of FGR-FBT hydroalcoholic extracts. Interestingly, the protective activity of the water extracts was shown to be as strong as the hydroalcoholic extracts at the same concentrations. In addition, there were significant differences (*P* < 0.05) in the DNA damage-protective activities of FGR-FBT and FGR. Scarce protective effects were determined at low concentrations (*P* > 0.05), while at high concentrations, FGR extracted by 80% ethanol showed a protective potential for supercoiled DNA exposure in Fenton reagents. Zhang et al. reported that catechin, epicatechin, and other phenolics were considered to have remarkable protective capabilities for the inhibition of DNA scission [36]. Also, it has been demonstrated in previous studies that DNA oxidative damage can be significantly inhibited by phenolic and flavonoid compounds via two mechanisms: chelating ferrous ions and scavenging H_2_O_2_/hydroxyl radicals [35,37]. In this study, the results of the DNA damage and protection assay obtained similar tendencies in antioxidant activity assays in vitro. This suggests that an increased phenolic content enhances the protective capability by fermented glutinous rice against DNA scission. Therefore, the supplementation of FBT is a crucial source of phenolics and FGR produced by fermentation with FBT may improve the chances of phenolics accumulation, thereby resulting in enhanced protective activities against oxidant-reduced diseases.

## 3. Materials and Methods

### 3.1. Materials

Fu brick tea (*Camellia sinensis* L.) was obtained from a dark tea factory in Yiyang city, Hunan province, China. The glutinous rice (*Oryza sativa* var.) was bought from a local supermarket.

The compounds 2,2-diphenyl-1-picrylhydrazyl (DPPH), 2,4,6-tris (2-pyridyl)-*S*-triazine (TPTZ), 2,2′-azino-bis-3-ethylbenzthiazoline-6-sulphonic acid (ABTS), ferrozine, ascorbic acid (vitamin C), 1,10-phenanthroline, and pUC18 plasmid DNA were purchased from Sigma-Aldrich Chemical Co. (St. Louis, MO, USA). The standards: gallic acid (GA), (−)-gallocatechin (GC), theobromine (Tb), 3,4-dihydroxybenzoic acid (DbA), theophylline (Tp), (−)-epigallocatechin (EGC), 3,4-dihydroxybenzaldehyde (Dbd), caffeine, catechin (C), caffeic acid (CA), (−)-epicatechin (EC), (−)-epigallocatechin gallate (EGCG), (−)-gallocatechin gallate (GCG), and (−)-epicatechin gallate (ECG) were purchased from Yuan-ye Biotech Co. (Shanghai, China). LC-grade methanol, acetonitrile, and trifluoroacetic acid were purchased from Merck (Darmstadt, Germany). Folin–Ciocalteu reagent and all other chemicals were analytical grade and purchased from Sinopharm Chemical Reagent Co. (Shanghai, China).

### 3.2. Solid-State Fermentation of Glutinous Rice

Sticky rice (200 g) was soaked with 800 mL deionized water at room temperature for 6 h, and then steamed for 30 min. After cooling to 30 °C, the steamed sticky rice was inoculated with commercial starters (Angel Yeast Co., Yichang, Hubei Province, China) at a ratio of 0.3 g of starters to 100 g of rice, to produce fermented glutinous rice. As for the novel fermented glutinous rice product with Fu brick tea (FGR-FBT), apart from commercial starters, FBT powder was mixed at different ratios of 0.5%, 1%, 2%, and 3%, respectively. FBT powder was milled and filtered before use, to that the particle size was less than 200 µm. The optimized proportion of FBT supplementation was determined by juice yield, fermented time, and sensory description, including color, appearance, aroma, taste, texture, and overall acceptability. The score of each index was calculated into a 9-score system, and a higher score indicated better properties. The supplementation proportion was finalized by a comprehensive analysis. FGR and FGR-FBT were brewed at 30 °C for 30 h, and its juice was obtained by extrusion in the terminal of fermentation.

### 3.3. Descriptive Sensory Analysis

The sensory description was performed by 10 panelists (five males and five females) and it was repeated in triplicate on different days. A three-hour training session was provided at the beginning for evaluating five different FGR products according to a modified aromatic scale [8,38], in which all the panelists had extensive experiences in describing their sensory evaluations for the FGR and FGR-FBT samples. A total of 16 descriptors of appearance (lightness, color, and turbidity), aroma/flavor (alcohol, acid, tea, and cereal), taste/mouth-feel (sweet, sour, bitter, astringency, aftertaste, and irritant) and basic texture (full body, granular feel, and continuation) were generated for characterizing the sensory properties of the fermented glutinous rice products. For each attribute, the score ranged from 0 to 9 in 0.1 increments (0: none; 1–2: very weak; 3–4: ordinary; 5–6: moderate; 7–8: strong; 9: very strong) [39]. All panelists were given FGR and FGR-FBT samples randomly, and were required to rinse their mouth with purified water before evaluating other samples. They were asked to finish the 16-parameter description at a rate of 15 min per sample. Besides, sensory scales of color, appearance, aroma, taste, texture, and overall acceptability were evaluated by using a quantitative descriptive analysis involving a 0–9 10-point interval scale for further statistical analysis (very bad: 0–2 scores; bad: 3–4 scores; normal: 5–6 scores; good: 7–8 scores; very good: 9 scores) [40].

### 3.4. Preparation of Extracts by Different Solvents

Previous studies have shown that water and 80% ethanol are efficient for the extraction of most phenolics from plant materials [26,27]. Thus, this study utilized these two solvents for the extraction of functional compounds in FGR and FGR-FBT [41]. One part of the samples was extracted by deionized water (1/50, *v*/*v*) in a boiling water bath for 5 min, while the others were extracted by 80% ethanol (1/50, *v*/*v*) at 50 °C for 20 min with ultrasonic assistance (300 W). The extracts were centrifuged at 15,000× *g* for 10 min at 4 °C to collect the supernatant. The residues were re-extracted twice by using the same methods. The extracted supernatant was combined and concentrated using a rotary evaporator (Heidolph Instruments Co., Ltd., Schwabach, Germany) under reduced pressure at 50 °C until dryness, before re-dissolving in the corresponding extract solvent. The re-dissolved extracts were filtered by a syringe filter (0.22 μm membrane, Bedford, MA, USA) and stored in the dark at 4 °C for further analysis.

### 3.5. Determination of Total Phenolic Contents

The total phenolic contents were determined using the Folin–Ciocalteu method [41]. Briefly, 0.2 mL of extract, 2.3 mL of deionized water, and 0.5 mL of Folin–Ciocalteu reagent were mixed with 2 mL of 7.5% Na_2_CO_3_ solution. The absorbance of the reaction mixture was read at 760 nm, using a spectrophotometer (Model 4001/4, Genesys 20 Thermo-Spectronic, Thermo Electron, Waltham, MA, U.S.A.) after incubation for 2 h at room temperature in the dark. A calibration curve was obtained using gallic acid as the standard and the total phenolic contents (TPC) of the samples were expressed as micrograms of gallic acid equivalents per gram of dry weight of extract (mg gallic acid/g extract).

### 3.6. High Performance Liquid Chromatography Analysis of Major Phenolic Compounds

Gallic acid, syringic acid, catechin, epicatechin, and other phenolics have been found in fermented glutinous rice by previous studies [18,20]. In this study, the concentrations of phenolic compounds in FGR were much lower than FGR-FBT according to our preliminary experiments. Thus, catechins and 14 other major chemicals were quantified by HPLC analysis to confirm the special functional compounds in FGR-FBT. All of the prepared extract solutions (see Section 2.5) were filtered and injected into a HPLC system (Agilent Technologies, Wilmington, DE, USA) equipped with a diode-array detector (DAD). Exactly 20 µL of each injected sample was isolated by a C18 HPLC reverse-phase column (4.6 mm × 250 mm, 5 mm particle size, Agilent Technologies, Wilmington, DE, USA) at 25 °C, and the compounds were identified by comparing their retention times with standards. The mobile phases consisted of solvent A (0.1% trifluoroacetic acid in deionized water) and solvent B (0.1% trifluoroacetic acid in acetonitrile) at a flow rate of 0.7 mL/min. A gradient programme was applied, beginning with a gradient of 5% B (95% A) at 0 min, increased to 6% B at 6 min, and then solvent B was linearly increased to 12% within 4 min, to 15% for 15 min, to 25% for 20 min, and to 35% for 7 min before returning to the initial ratio for 8 min. UV detection was performed at the wavelength of 280 nm. The concentrations of the identified compounds were expressed as micrograms per gram of dry weight of extract (mg/g extract).

### 3.7. Antioxidant Activities Evaluation

#### 3.7.1. Assay for DPPH Radical-Scavenging Activity

The DPPH radical-scavenging activity was studied according to the method of Wang et al. [14] as follows: 2 mL of test solution was mixed with 2 mL of DPPH solution (0.2 mM), and then incubated at room temperature for 30 min in the dark. The absorbance was recorded at 517 nm (GENESYS 20 Thermo-Spectronic, Thermo Electron Corp., Waltham, MA, USA). The activity of the DPPH radical-scavenging was expressed by the scavenging ratio, and calculated by the following Equation (1):

DPPH radical-scavenging ratio (%) = [1 − A_sample_/A_control_] × 100 (1)

in which A_sample_ is the absorbance with the test sample, and A_control_ is the absorbance with extract solvents instead of samples in the assay system (containing all reagents except the test samples). Ascorbic acid (Vitamin C, V_C_) was used as a positive control.

#### 3.7.2. Assay for ABTS·^+^-Scavenging Activity

The ABTS radical cation (ABTS·^+^)-scavenging activity was analyzed in a micro-system according to the method of Xiao et al. [26]. ABTS·^+^ was generated by mixing the ABTS solution (7 mM) with a 2.45 mM aqueous solution of K_2_S_2_O_8_ at room temperature for 16 h in the dark. It was diluted with ethanol before using a ABTS·^+^ reaction solution whose absorbance was 0.70 ± 0.02 at 734 nm. Volume 200 μL of samples and 800 μL of ABTS·^+^ reaction solution were mixed and reacted for 6 min at room temperature. The absorbance was recorded at 734 nm. The capability of scavenging ABTS·^+^ was expressed as scavenging ratio by calculation, based on the following Equation (2):

ABTS·^+^ scavenging ratio (%) = [1 − A_sample_/A_control_] × 100 (2)

where A_control_ is the absorbance with extract solvents instead of the test samples, and A_sample_ is the absorbance with test samples in the assay system. V_C_ was used as a positive control.

#### 3.7.3. Ferric-Reducing Antioxidant Power (FRAP) Assay

The FRAP assay was modified based on the method studied by Xiao et al. [26]. The fresh FRAP reaction solution was prepared by mixing 10 mL TPTZ (10 mM), 10 mL ferric chloride (20 mM), and 100 mL acetate buffer (0.3 M, pH 3.6). Volumes of 200 μL test samples and 1000 μL FRAP reaction solution were mixed and incubated at 37 °C for 20 min in the dark. The absorbance was recorded at 593 nm. A standard curve equation as follows was obtained based on FRAP assays with different concentrations of ferrous sulphate (0.4–2.4 mM). The results were expressed as micromoles of Fe(II) equivalents per gram of dry weight sample (3):

y = 0.0022x − 0.009 (R^2^ = 0.9998) (3)

#### 3.7.4. Reducing Power Assay

The reducing power was estimated according to the method of Zhai et al. [42]. The reaction solution was mixed with 0.5 mL volumes of samples, 2.5 mL of PBS (0.2 M, pH 6.6) and 2.5 mL of potassium ferricyanide (1%, *w*/*v*), and incubated at 50 °C for 20 min, followed by the addition of 2.5 mL of trichloroacetic acid (10%, *w*/*v*) to terminate the reaction. Then, the reaction solution was centrifuged at 450 g for 10 min and 2.5 mL of the supernatant was collected to mix with 0.5 mL of ferric chloride (0.1%, *w*/*v*). After 10 min of reaction was executed at room temperature, the absorbance was recorded at 700 nm. A higher absorbance indicated a higher reducing power. V_C_ was used as a positive control.

#### 3.7.5. Evaluation of Ferrous Ion Chelating Activity

The chelating activity on ferrous ion was conducted according to the study of Wang et al. [19]. In brief, 1 mL of sample was mixed with 3.7 mL of deionized water, 0.1 mL of ferrous chloride (2 mM) and 0.2 mL of ferrozine (5 mM). After 20 min of reaction at room temperature, the absorbance was recorded at 562 nm. The results were expressed by chelating ratio, and calculated by the following Equation (4):

Ferrous ion chelating ratio (%) = [1 − A_sample_/A_control_] × 100 (4)

where A_control_ was the absorbance with extract solvents instead of test samples, and A_sample_ was the absorbance with test samples. Ethylene diamine tetraacetic acid was used as a positive control.

#### 3.7.6. Hydroxyl Radical-Scavenging Assay

The hydroxyl radical-scavenging activity was determined using the method of Li et al. [43]. Briefly, hydroxyl radicals were generated by mixing 1 mL of FeSO_4_ (0.75 mM), 1 mL of 1,10-phenanthroline (0.75 mM), 1 mL of H_2_O_2_ (0.01%, *v*/*v*) and 1.5 mL of sodium phosphate buffer (0.15 M, pH 7.4). Then, 1 mL of sample was mixed with above reaction solvent and incubated at 37 °C for 30 min. The absorbance was read at 536 nm. The result was expressed by the scavenging ratio, and calculated based on the following Equation (5):

Hydroxyl radical-scavenging ratio (%) = (A_sample_ − A_blank_)/(A_0_ − A_blank_) × 100 (5)

where A_0_ is the absorbance with extract solvent and deionized water, instead of the sample and H_2_O_2_ (containing all reagents except test samples and H_2_O_2_), A_sample_ and A_blank_ were the absorbance with test samples and deionized water, respectively, in the assay system. V_C_ was used as a positive control.

### 3.8. Assessment of Supercoiled Plasmid DNA Strand Breakage Inhibition

To evaluate the protective capabilities of samples against DNA damage, extracts of FGR-FBT and FGR were diluted into gradient concentrations and analyzed based on the method of Xiao et al. [27]. Fenton’s reagent was prepared with FeCl_3_ (80 mM), ascorbic acid (50 mM), and H_2_O_2_ (30 mM). Volumes of 10 μL sample and 1 μL of pUC18 plasmid DNA (200 ng/mL) were mixed, and then 10 μL of Fenton’s reagent was added. After 30 min of reaction at 37 °C, the DNA in each assay system was electrophoresed in a 1% (*w*/*v*) agarose gel for 40 min under 120 V conditions. The gel was visualized under a UV-transilluminator using the Gel Doc XR system (Bio-Rad, Hercules, CA, USA). The optical intensities of the DNA bands were analyzed by using Quantity One software (version 4.6.2, BioRad). Phosphate buffer saline was used to replace the samples as a negative control, and to replace Fenton’s reagent as a blank control. The protective capability was expressed as a percentage of supercoiled DNA, which was calculated using the following Equation (6):

Supercoiled DNA (%) = [A_s_/(A_s_ + A_o_)] × 100 (6)

where A_s_ is the optical intensity of normal DNA (supercoiled DNA form) and A_o_ is the optical intensity of oxidatively damaged DNA (open circular form). A higher supercoiled DNA ratio indicates a higher protective activity for damaged DNA.

### 3.9. Statistical Analysis

All of the results in this study are presented as means ± standard deviation. One-way analysis of variance (ANOVA), Duncan’s multiple range tests, and T-tests were carried out to analyze significant differences (*p* < 0.05), using SPSS version 17.0 (SPSS Inc., Chicago, IL, USA). The IC_50_ values were obtained by interpolation or extrapolation from linear regression analysis, according to the previous study by Xiao et al. [27].

## 4. Conclusions

In this study, a novel functional food, fermented glutinous rice with Fu brick tea, was developed based on fermented glutinous rice with the supplementation of Fu brick tea (FBT). FBT increased the total phenolic content, elevated the aromatic and taste properties, and enhanced the antioxidant activities of traditional fermented glutinous rice, including free radical-scavenging activities, reducing power, and chelating activities for metal ions. Additionally, this study was the first to report the DNA protective ability of glutinous rice products. Compared with water extracts, 80% ethanol extracted more functional compounds, and thus it may be utilized as an extract solvent to obtain nutrition-rich ingredients. In total, these findings are of great interest because the antioxidant activity of glutinous rice is increased by fermentation with FBT, and also facilitated the applications of FBT. Therefore, this novel product could be consumed as a phenolic-rich functional staple food or a nutraceutical food additive.

## Figures and Tables

**Table 1:** Sensory evaluation of fermented glutinous rice with different proportions of the supplementation of Fu brick tea (FGR-FBT) and traditional fermented glutinous rice (FGR).

		Control	FGR-0.5%FBT	FGR-1%FBT	FGR-2%FBT	FGR-3%FBT
**Appearance**	Lightness	9.00 ± 0.00 ^a^	7.30 ± 0.56 ^b^	6.85 ± 0.59 ^bc^	6.35 ± 0.81 ^c^	5.65 ± 0.84 ^d^
	Color	2.00 ± 0.63 ^d^	4.75 ± 0.87 ^c^	6.15 ± 0.59 ^b^	7.15 ± 0.59 ^a^	7.80 ± 0.64 ^a^
	Turbidity	6.90 ± 0.49 ^a^	7.05 ± 0.52 ^a^	7.10 ± 0.66 ^a^	7.40 ± 0.44 ^a^	7.20 ± 0.56 ^a^
**Aroma/flavor**	Alcohol	8.10 ± 0.37 ^a^	7.85 ± 0.55 ^ab^	7.75 ± 0.46 ^ab^	7.70 ± 0.46 ^ab^	7.40 ± 0.62 ^b^
	Acid	7.75 ± 0.40 ^a^	7.50 ± 0.55 ^ab^	7.10 ± 0.66 ^bc^	6.65 ± 0.74 ^c^	5.55 ± 0.72 ^d^
	Tea	0.00 ± 0.00 ^e^	5.35 ± 0.90 ^d^	6.90 ± 0.58 ^c^	7.55 ± 0.52 ^b^	8.30 ± 0.40 ^a^
	Cereal	8.30 ± 0.40 ^a^	8.35 ± 0.39 ^a^	7.30 ± 0.51 ^b^	6.40 ± 0.70 ^c^	5.20 ± 0.98 ^d^
**Taste/mouth-feel**	Sweet	8.05 ± 0.27 ^a^	7.80 ± 0.46 ^ab^	7.25 ± 0.46 ^b^	6.15 ± 0.71 ^c^	4.90 ± 0.83 ^d^
	Sour	6.95 ± 0.52 ^a^	6.60 ± 0.54 ^ab^	6.50 ± 0.45 ^ab^	6.25 ± 0.46 ^bc^	5.95 ± 0.52 ^c^
	Bitter	0.75 ± 0.60 ^d^	1.30 ± 0.46 ^d^	3.35 ± 0.63 ^c^	4.45 ± 0.57 ^b^	6.40 ± 0.92 ^a^
	Astringency	2.60 ± 0.54 ^d^	2.80 ± 0.75 ^d^	3.80 ± 0.75 ^c^	4.90 ± 0.83 ^b^	6.65 ± 0.71 ^a^
	Aftertaste	3.95 ± 0.72 ^e^	5.00 ± 0.67 ^d^	5.80 ± 0.78 ^c^	7.25 ± 0.75 ^a^	6.40 ± 0.54 ^b^
	Irritant	5.45 ± 0.61 ^a^	5.25 ± 0.81 ^a^	5.00 ± 0.63 ^a^	4.90 ± 0.50 ^a^	4.70 ± 0.51 ^a^
**Basic texture**	Full body	7.30 ± 0.40 ^a^	7.10 ± 0.49 ^ab^	7.00 ± 0.50 ^ab^	6.70 ± 0.40 ^bc^	6.30 ± 0.40 ^c^
	Granular sensor	1.20 ± 0.33 ^d^	1.55 ± 0.47 ^d^	3.50 ± 0.45 ^c^	4.65 ± 0.63 ^b^	6.50 ± 0.84 ^a^
	Continuation	4.25 ± 0.46 ^c^	4.55 ± 0.47 ^c^	5.60 ± 0.54 ^b^	7.40 ± 0.77 ^a^	7.05 ± 0.73 ^a^

Each value was expressed as the mean ± standard deviation (n = 10). Means with different lowercase letters (a, b, c, d, and e) within a row indicated significant differences (*p* < 0.05).

**Table 2:** Major functional compounds of water and hydroalcoholic extracts (80% ethanol extracts) of fermented glutinous rice with Fu brick tea (FGR-FBT).

	Compounds	FGR-FBT	
		Water Extracts	Hydroalcoholic Extracts
1	GA	77.47 ± 0.33 ^b^	81.30 ± 0.31 ^a^
2	GC	255.97 ± 1.71 ^b^	269.81 ± 2.77 ^a^
3	Tb	9.80 ± 0.07 ^a^	4.64 ± 0.17 ^b^
4	DbA	2.62 ± 0.12 ^a^	2.81 ± 0.13 ^a^
5	Tp	1.42 ± 0.02 ^a^	1.43 ± 0.03 ^a^
6	EGC	28.95 ± 0.14 ^b^	38.88 ± 0.72 ^a^
7	Dbd	13.18 ± 0.04 ^a^	13.25 ± 0.10 ^a^
8	Caffeine	272.46 ± 0.13 ^a^	267.06 ± 0.54 ^b^
9	C	9.09 ± 0.20 ^b^	26.35 ± 1.15 ^a^
10	CA	0.46 ± 0.03 ^a^	0.26 ± 0.02 ^b^
11	EC	119.71 ± 1.72 ^b^	152.98 ± 1.41 ^a^
12	EGCG	46.31 ± 0.17 ^b^	48.76 ± 0.39 ^a^
13	GCG	41.81 ± 0.06 ^b^	45.85 ± 0.67 ^a^
14	ECG	7.76 ± 0.07 ^b^	8.31 ± 0.22 ^a^

GA: gallic acid, GC: (−)-gallocatechin, Tb: theobromine, 3, DbA: 4-dihydroxybenzoic acid, Tp: theophylline, EGC: (−)-epigallocatechin, Dbd: 3,4-dihydroxybenzaldehyde, C: catechin, CA: caffeic acid, EC: (−)-Epicatechin, EGCG: (−)-epigallocatechin gallate, GCG: (−)-gallocatechin gallate, ECG: (−)-epicatechin gallate. Each value is expressed as the mean ± standard deviation (n = 3). Means with different lowercase letters (a and b) within a row indicate significant differences (*p* < 0.05).

**Table 3:** IC_50_ values in antioxidant properties of water and hydroalcoholic extracts (80% ethanol extracts) of fermented glutinous rice with Fu brick tea (FGR-FBT) and traditional fermented glutinous rice (FGR).

	FGR-FBT		FGR	
	Water Extracts	Hydroalcoholic Extracts	Water Extracts	Hydroalcoholic Extracts
ABTS·^+^	30.67 ± 0.29 ^c^	10.75 ± 0.24 ^d^	257.71 ± 4.82 ^a^	224.55 ± 3.63 ^b^
DPPH	17.26 ± 0.30 ^c^	15.97 ± 0.21 ^d^	132.75 ± 3.09 ^a^	114.83 ± 4.43 ^b^
FRAP	26.61 ± 2.43 ^c^	13.23 ± 1.87 ^d^	351.67 ± 26.62 ^a^	319.04 ± 32.92 ^b^
Reducing power	265.61 ± 10.69 ^b^	126.42 ± 3.83 ^c^	1654.89 ± 24.26 ^a^	1648.89 ± 8.19 ^a^
Fe^2+^ chelating *	498.43 ± 13.75 ^d^	705.47 ± 55.92 ^c^	1679.88 ± 67.05 ^b^	1893.39 ± 64.13 ^a^
·OH *	969.79 ± 17.49 ^a^	292.19 ± 4.03 ^b^	nd	nd

IC_50_ value: the effective concentration at which the radical is scavenged by 50% or where absorbance is 0.5 for reducing power. IC_50_ values were obtained by interpolation from linear regression analysis. * indicates that the IC_50_ values of Fe^2+^-chelating capability and hydroxyl radical-scavenging activities were calculated by extrapolation from linear regression analysis. “nd” indicates that no IC_50_ values could be calculated. Different lowercase letters (a, b, c and d) within a row indicate significant differences (*p* < 0.05).
